# Thermally Enhanced Dual-Mode Resistive Switching via Interfacial Strain Engineering in SmNiO_3_/PMN-PT Heterostructures

**DOI:** 10.3390/ma19183838

**Published:** 2026-09-09

**Authors:** Jianfeng Yang, Qin Du

**Affiliations:** Shaanxi Key Laboratory of Intelligent Processing for Big Energy Data, School of Physics and Electronic Information, Yan’an University, Yan’an 716000, China; yangjf@yau.edu.cn

**Keywords:** SmNiO_3_ thin films, oxide heterostructures, interfacial strain engineering, resistive switching, ferroelastic domain switching

## Abstract

**Highlights:**

- High-quality SmNiO_3_ films are stabilized on PMN-PT using a LaAlO_3_/SrTiO_3_ buffer layer.
- Dual-mode (volatile and nonvolatile) resistive switching is achieved in SmNiO_3_/PMN-PT.
- Thermally boosted modulation (−6.4% → −11.9% at 75 °C at same bias).
- The strain-mediated mechanism is demonstrated as a proof-of-concept for electronic applications.

**Abstract:**

SmNiO_3_ thin films on PMN-PT ferroelectric substrates enable electrically switchable strain engineering, an advantage over permanently fixed lattice-mismatch strain. However, strain-engineered resistive switching in SmNiO_3_ via ferroelectric substrates remains largely unexplored. Here, by stabilizing high-quality epitaxial SmNiO_3_ films on (011)-cut PMN-PT through a LaAlO_3_/SrTiO_3_ graded buffer interface, we demonstrate dual-mode resistive modulation in SmNiO_3_/PMN-PT heterostructures: reversible butterfly-shaped hysteresis under bipolar fields arising from dynamic electrostrain, and nonvolatile switching through non-180° ferroelastic domain reorientation. Moderate heating to 75 °C boosts the resistance modulation, correlated with enhanced strain output from the PMN-PT substrate near its phase transition. At the same bias of 12 kV/cm, the modulation increases from −6.4% at 25 °C to −11.9%; even at only 6 kV/cm, the modulation reaches −6.7%, exceeding the room-temperature value at 12 kV/cm. These results demonstrate the potential of SmNiO_3_/PMN-PT heterostructures for dual-mode resistive switching, integrating both volatile and nonvolatile modulation within a single material system through a thermally enhanced strain-coupling mechanism. This work serves as a proof-of-concept demonstration for future exploration in neuromorphic applications.

## 1. Introduction

Rare-earth nickelates ReNiO_3_ exhibit rich electronic phases under external stimuli such as temperature, hydrogenation, electric field, and stress, making them attractive for applications in resistive sensing, artificial computing, energy conversion, and weak-field detection [1,2,3]. The complexity of their electronic behavior originates from the orbital configurations dictated by NiO_6_ octahedral distortions. The strong coupling between lattice and orbital degrees of freedom renders stress an effective tuning knob for the Ni-3d and O-2p hybridization, which governs the electronic transport properties [4]. In epitaxial heterostructures, lattice mismatch between the film and substrate generates interfacial strain that modifies the orbital overlap and thus the resistivity [5], but this static strain cannot be dynamically controlled, limiting its use in reconfigurable electronics. In contrast, ferroelectric single-crystal substrates with excellent piezoelectric properties enable dynamic, electrically controllable strain in the overlying correlated oxide films (e.g., VO_2_, Nd_0.5_Sr_0.5_MnO_3_, and L_a0.33_Ca_0.67_MnO_3_) [6,7,8,9,10]. This approach offers a promising route toward strain-mediated resistive switching devices [11,12].

In early attempts to control the resistivity of NdNiO_3_ (NNO) using ferroelectric substrates, a 0.25% in-plane compressive strain shifted the metal-insulator transition temperature (*T*_MI_) by merely ~3.3 K, resulting in limited resistance modulation at fixed temperatures [13]. Subsequent work leveraging ferroelastic domain switching in 0.31Pb(In_1/2_Nb_1/2_)O_3_-0.35Pb(Mg_1/3_Nb_2/3_)O_3_-0.34PbTiO_3_ (PIN-PMN-PT) substrates achieved substantially larger electrically induced in-plane tensile strains of +0.81%, leading to a resistance modulation as high as 125% at 200 K in NNO films [14]. The resulting multiple stable resistance states, with excellent retention and endurance, demonstrate the potential of ferroelectric domain engineering for multistate memristors.

Despite these advances, the strain-controlled resistive switching approach based on NNO faces fundamental limitations, including a cryogenic *T*_MI_ and a narrow operating window near the transition critical point, whereas alternative strategies involving ionic doping introduce separate electrochemical concerns [15,16]. These limitations have motivated the search for alternative nickelate systems that can offer room-temperature operation, strain-mediated control, and integration of both volatile and nonvolatile resistive switching functions within a single material platform.

In this work, we address these challenges by investigating electric-field-controlled strain engineering in high-quality epitaxial SmNiO_3_ (SNO) thin films grown on (011)-cut 0.70Pb(Mg_1/3_Nb_2/3_)O_3_-0.30PbTiO_3_ (PMN-PT) substrates. Unlike NNO, SNO exhibits a *T*_MI_ well above room temperature, allowing the system to remain insulating under ambient conditions and enabling room-temperature strain-mediated resistive switching. More importantly, we demonstrate that the SNO/PMN-PT heterostructure integrates both reversible (butterfly-shaped) and nonvolatile (hysteretic) resistive switching, arising from dynamic electrostrain and non-180° ferroelastic domain reorientation, respectively. In addition, we reveal a thermally boosted modulation effect that significantly enhances the resistive switching performance at moderate temperatures. Our results suggest that SNO is a promising system for room-temperature strain-controlled resistive switching, with potential for future development in reconfigurable electronic applications.

## 2. Materials and Methods

PMN-PT single-crystal substrates with dimensions of 5 × 4 × 0.3 mm^3^ were purchased from the Shanghai Institute of Ceramics, Chinese Academy of Sciences (Shanghai, China). For the heterostructures, SrTiO_3_ (STO, a = 3.905 Å) and LaAlO_3_ (LAO, a = 3.799 Å) buffer layers were sequentially deposited on (011)-cut PMN-PT substrates by pulsed laser deposition (PLD) using a KrF excimer laser (λ = 248 nm; Coherent, Inc., Santa Clara, CA, USA). The STO layer was deposited first, followed by the LAO layer. Both layers were grown at 700 °C under 13 Pa O_2_, with a laser energy density of ~4 J/cm^2^, a repetition rate of 2 Hz, and a target–substrate distance of 6.5 cm. The individual buffer layer thicknesses were calibrated by cross-sectional scanning electron microscopy (SEM; TESCAN MAIA3, TESCAN, Brno, Czech Republic) (Figure S1) to be approximately 5 nm each.

SmNiO_3_ (SNO) thin films were then grown on the buffered PMN-PT substrates, as well as one-side-polished (001)-oriented LAO and STO single-crystal substrates (Hefei Kejing Materials Technology Co., Ltd., Hefei, China) by PLD. The deposition was performed at 700 °C under 20 Pa O_2_, with a laser energy density of ~4 J/cm^2^, a repetition rate of 2 Hz, and a target–substrate distance of 6.5 cm. After deposition, the films were annealed in situ at 700 °C for 20 min and then cooled to room temperature at 20 °C/min. The SNO film thickness was approximately 100 nm.

The crystallinity and phase purity of the films were characterized by X-ray diffraction (XRD) *θ*–2*θ* scans using a Philips X’Pert high-resolution diffractometer (Empyrean, Malvern Panalytical B.V., Almelo, The Netherlands) with Cu Kα1 radiation (λ = 1.5406 Å). For electrical measurements, circular Pt top electrodes (~200 μm diameter) were deposited onto the SNO films through a shadow mask by magnetron sputtering (Phase II, AJA International, Inc., Hingham, MA, USA) at room temperature, and Au bottom electrodes were sputtered onto the back sides of the PMN-PT substrates. Electric fields were applied across the SNO/PMN-PT heterostructures using a Keithley 6517B voltage source (Keithley Instruments, Cleveland, OH, USA). The in-plane resistance of the SNO films was measured in a two-terminal configuration with a probe spacing of approximately 300 μm using a Keithley 2700 multimeter (Keithley Instruments, Cleveland, OH, USA) at a constant current of 100 µA. The leakage current (~10^−8^ A) is negligible compared to the resistance measurement current (100 µA), ruling out leakage-related artifacts. The electric-field-induced strain (*ε*–*E*) curves of the PMN-PT substrates were measured using a TF Analyzer 2000 ferroelectric measurement system (aixACCT Systems GmbH, Aachen, Germany).

## 3. Results and Discussion

### 3.1. Epitaxial Growth and Structural Quality

**Figure 1:**
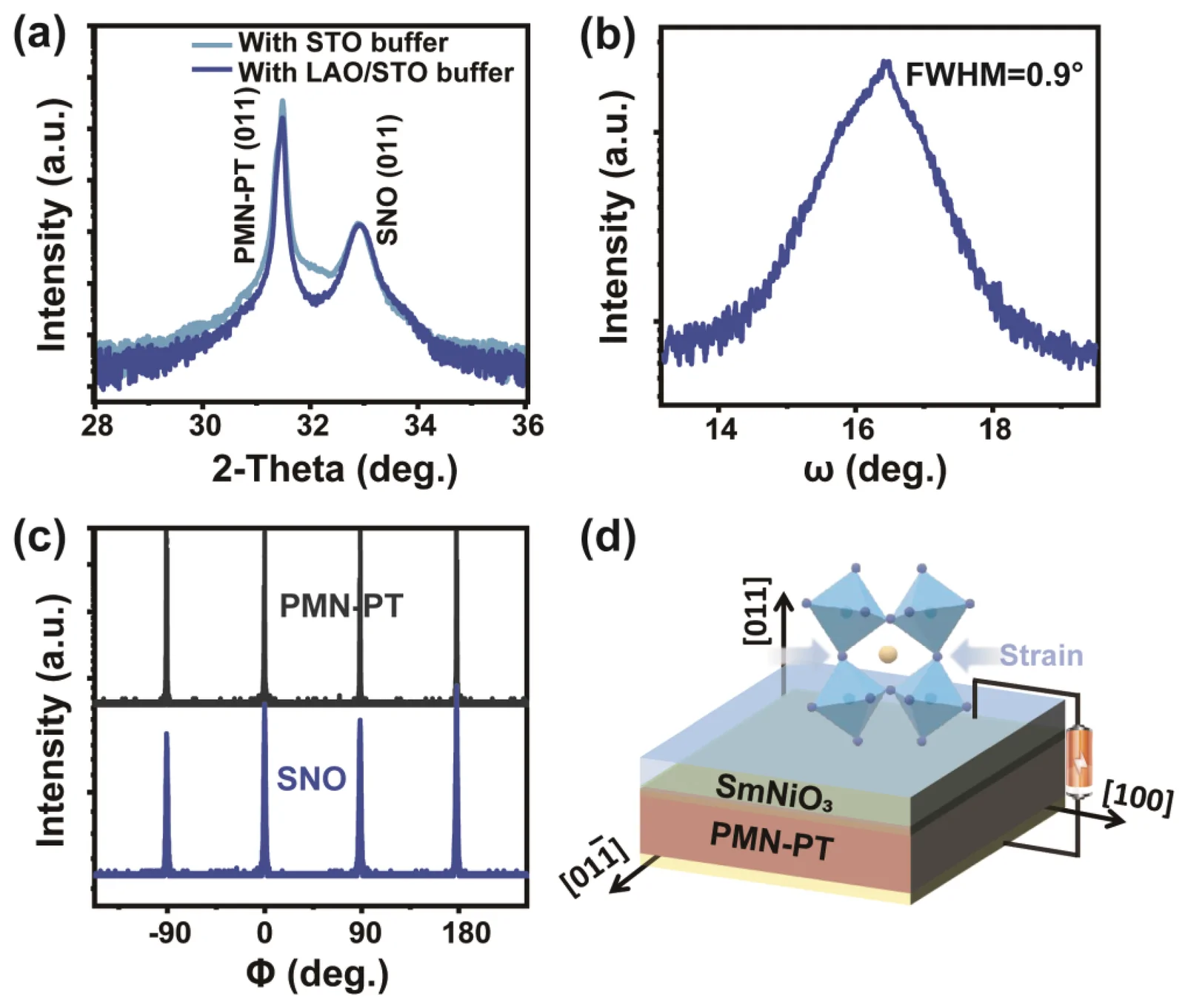
(**a**) XRD *θ*–2*θ* patterns of SNO films deposited on STO-buffered and LAO/STO-buffered (011)-cut PMN-PT substrates. (**b**) XRD rocking curve of the SNO (011) reflection for the SNO/PMN-PT heterostructure with LAO/STO buffer layers. (**c**) XRD φ-scans of the (101) reflection for SNO and PMN-PT. (**d**) Schematic illustration of the in-plane strain in the SNO film tuned via ferroelectric polarization of the PMN-PT substrate.

Epitaxial growth on appropriately buffered ferroelectric substrates is critical for achieving high-quality correlated oxide films with well-controlled strain states. The STO and LAO/STO interlayers served as structural templates for heteroepitaxial growth, leveraging their closer lattice match with SNO to reduce the nucleation energy barrier and improve crystalline quality. The symmetric *θ*–2*θ* XRD scans in Figure 1a show only the (011) diffraction peak of SNO, indicating preferential out-of-plane orientation of the film. Notably, the SNO film grown on LAO/STO-buffered PMN-PT substrate exhibited a sharper (011) peak compared with that on STO-buffered substrate, suggesting improved crystalline quality. The rocking curve (ω-scan) for the SNO (011) reflection (Figure 1b) yields a full width at half maximum (FWHM) of 0.9°, further supporting the high-quality epitaxial growth enabled by the graded buffer structure. In addition, the φ-scan patterns of the SNO film (Figure 1c) exhibit peaks with four-fold symmetry, matching the symmetry of the underlying PMN-PT (011) substrate. This demonstrates a clear epitaxial relationship between the film and the substrate. Unless otherwise specified, all SNO samples used in subsequent measurements refer to those grown on LAO/STO-buffered PMN-PT substrates (hereafter denoted as SNO/PMN-PT).

Figure 1d schematically illustrates the in-plane strain of the SNO film tuned by the ferroelectric response of the PMN-PT substrate. The electronic transport properties of the SNO films were modulated in situ by poling the PMN-PT substrate along the [011] crystallographic direction. Since the SNO film is far more conductive than the PMN-PT substrate and the buffer layers are only a few nanometers thick, the applied electric field drops predominantly across the PMN-PT substrate.

**Figure 2:**
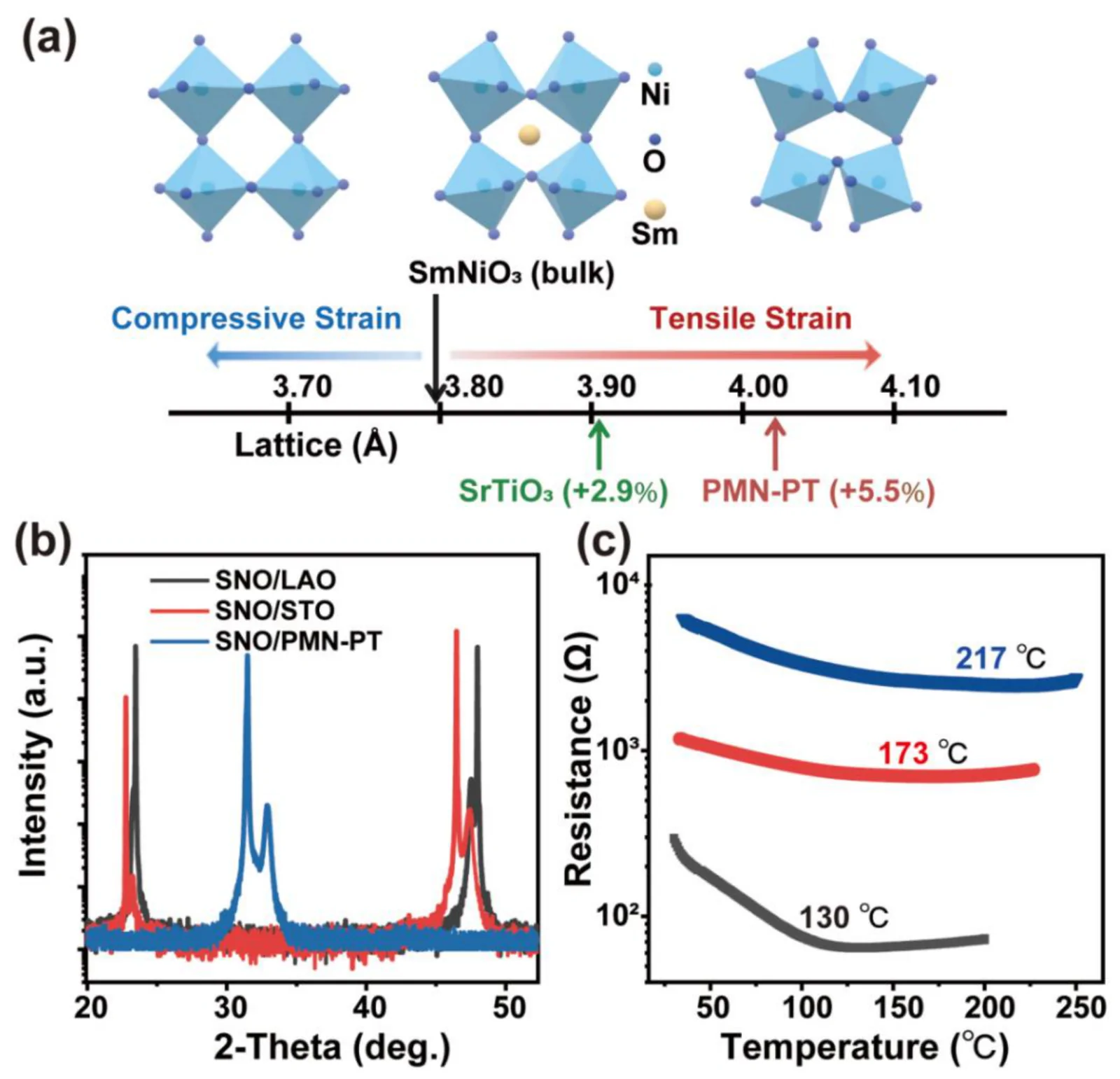
(**a**) Schematic illustration of the electronic structure modulation in SNO films via distortion of the NiO_6_ octahedra induced by interfacial strain. (**b**) XRD patterns of SNO films deposited on LAO, STO, and PMN-PT substrates. (**c**) Temperature-dependent resistance (*R*–*T*) for SNO films; the metal-insulator transition temperature (*T*_MI_) is indicated.

The electronic structures of SNO films are strongly modified by the epitaxial strain through distortion of the NiO_6_ octahedron (Figure 2a). To better understand the lattice strain effects, we also grew SNO films on (001)-oriented LAO and STO single-crystal substrates. The XRD *θ*–2*θ* patterns in Figure 2b show the (001) (2*θ* ≈ 23.3°), (002) (2*θ* ≈ 47.7°), and (011) (2*θ* ≈ 32.9°) Bragg peaks from SNO, along with substrate reflections. The absence of any non-epitaxial diffraction features confirms the out-of-plane alignment of the SNO films. The *T*_MI_ values were extracted from the *R*–*T* curves measured during warming at ~10 °C/min (Figure 2c), defined as the minimum of the first derivative (d*R*/d*T*). The SNO film grown on LAO substrate exhibited a *T*_MI_ of approximately 130 °C, consistent with reported values [1]. By contrast, the SNO films on STO and PMN-PT substrates, which have larger lattice mismatches with SNO (*a*_pc_ = 0.3905 nm for STO and 0.4022 nm for PMN-PT, compared with 0.3799 nm for SNO), exhibited broader transitions with *T*_MI_ values of approximately 173 °C and 217 °C, respectively. The large tensile strains in these films are responsible for the broadening and the upward shift in the *T*_MI_ [17].

### 3.2. Dual-Mode Resistive Switching: Volatile and Nonvolatile Modulation

**Figure 3:**
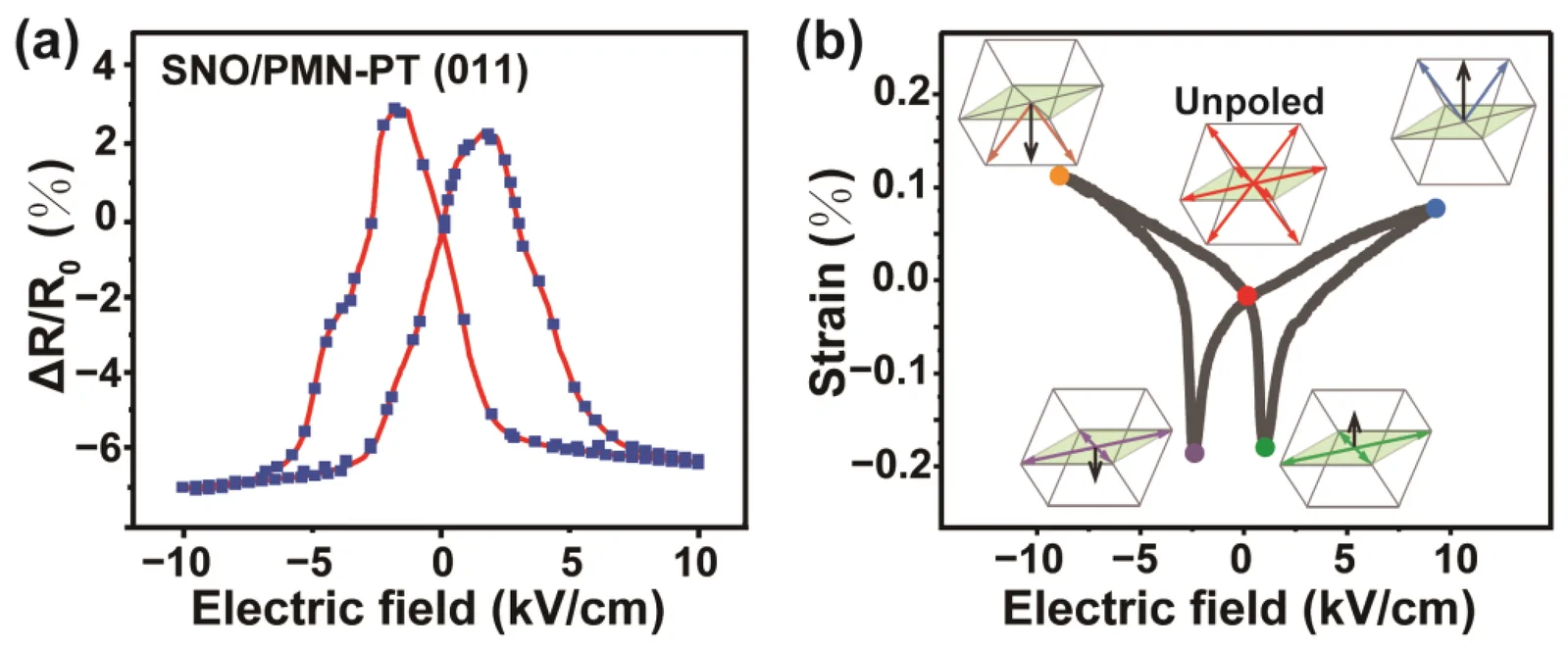
(**a**) Butterfly-shaped relative resistance change (Δ*R*/*R*_0_) of the SNO film as a function of bipolar electric field at room temperature. (**b**) Electric-field-induced strain (*ε*–*E*) curves along the [011] direction of the PMN-PT substrate; inset: schematic of the polarization states of rhombohedral PMN-PT under different electric fields, where the black arrows indicate the direction and magnitude of the applied electric field. The colored polarization vectors correspond to the different stages of the strain curve, with the same color code used for the corresponding data points in the *ε*–*E* curve.

The electric-field modulation of the SNO resistance was characterized at room temperature in a configuration where a bias voltage was applied across the thickness of the heterostructure (schematically shown in Figure S2), with the Pt/SNO stack serving as the top electrode and Au on the backside of the PMN-PT substrate as the bottom electrode. The in-plane resistance of the SNO film was measured in a two-terminal configuration using a probe spacing of approximately 300 μm. A Keithley 2700 multimeter with a constant current of 100 µA was used for resistance measurement, and the resistance was recorded during the voltage pulse (i.e., in situ under applied bias). Figure 3a shows the resistance response of the SNO film as a function of a bipolar electric field at room temperature. Here, Δ*R*/*R*_0_ = [*R*(*E*) − *R*(0)]/*R*(0), where *R*(*E*) and *R*(0) are the resistances under an applied field *E* and at zero field, respectively. A butterfly-shaped resistance–electric field (Δ*R*/*R*_0_–*E*) hysteresis loop is observed upon cycling a bipolar electric field with an amplitude of 10 kV/cm (red curve in Figure 3a). The resistance modulation reaches −6.2% at +10 kV/cm and −7.1% at −10 kV/cm. Two resistance peaks appear near the coercive field (*E*_c_ ≈ ±2 kV/cm), corresponding to the ferroelectric polarization switching between in-plane and out-of-plane orientations, which modulates the in-plane strain state of the PMN-PT substrate and thus the resistance of the SNO film [12]. The evolution of resistance closely follows the butterfly-shaped strain–electric field (*ε*–*E*) curve of the PMN-PT substrate at room temperature (Figure 3b), indicating that the resistance change in the SNO film is predominantly driven by the lattice strain transferred from the substrate. Alternative mechanisms such as Joule heating, electrostatic gating, charge injection, or oxygen-vacancy migration are unlikely to be the dominant contributors based on the following observations: the butterfly-shaped hysteresis tracks the strain curve with peaks at the coercive fields, the modulation is reversible and symmetric without cumulative drift, and the leakage current (limit: 50 µA) remains negligible compared to the measurement current (100 µA). To distinguish between strain-mediated and charge-mediated effects, we measured the polarization-vs.-electric field (*P*–*E*) loop of the PMN-PT (011) substrate (Figure S3). The loop exhibits a rectangular/square-like hysteresis with a coercive field of ~2 kV/cm, consistent with the resistance peaks in Figure 3a, supporting that the resistance change is predominantly strain-mediated rather than charge-mediated.

The inset of Figure 3b schematically illustrates the polarization states of the rhombohedral PMN-PT (011) substrate under different electric fields. In the initial state (zero field), the spontaneous polarization vectors (red arrows) lie along the eight equivalent <111> directions of the pseudo-cubic unit cell. Among them, four vectors are oriented within the (011) plane, with two pointing upward and two pointing downward relative to the plane. Upon application of a symmetric bipolar electric field (amplitude of 10 kV/cm), the *ε*–*E* curve exhibits a nearly symmetric strain response, with a volatile strain state retained at zero field. When the electric field exceeds the coercive field again, the polarization switches to the out-of-plane direction [18]. This process is accompanied by the transformation of the in-plane strain from tensile to compressive. The resulting compressive strain transferred to the SNO film reduces electronic localization and narrows the band gap, leading to a decrease in resistance as the electric field increases from the coercive field toward the positive or negative maximum.

**Figure 4:**
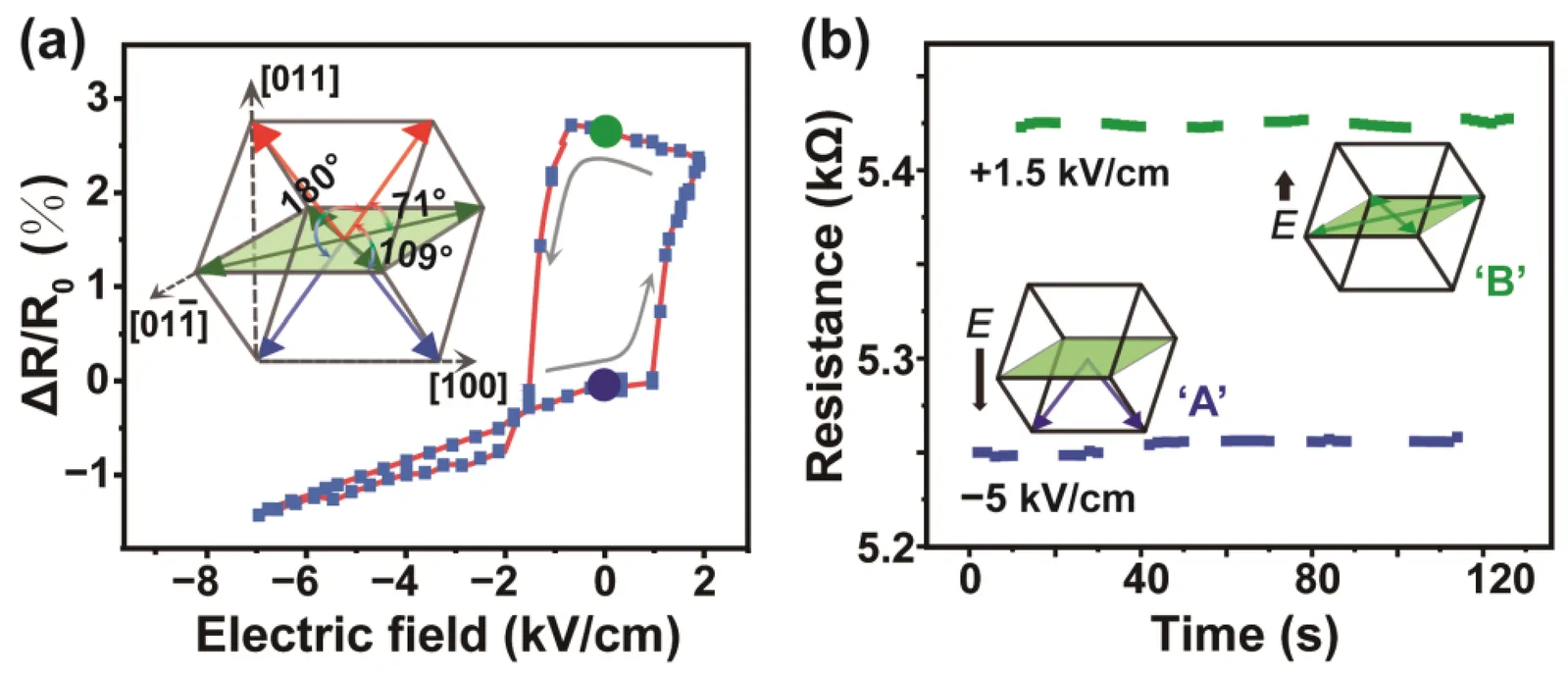
(**a**) Hysteretic resistance response under asymmetric electric field sweeping; inset: schematic of eight possible <111> polarization directions for rhombohedral PMN-PT and the 71°, 109°, and 180° domain switching paths. The colored polarization vectors in the inset of (**a**) correspond to the different resistance states, with the same color code used in (**b**) to indicate the corresponding resistive states. (**b**) Repeatability test of resistance switching under pulsed electric fields (pulse duration: 1 s); inset: two remanent polarization states of PMN-PT (out-of-plane state ‘A’ and in-plane state ‘B’).

However, when a bipolar electric field with asymmetric amplitudes is applied, the asymmetric polarization switching gives rise to a hysteretic resistance–voltage response (Figure 4a). During the negative sweep from 0 to −7 kV/cm, the field exceeds the coercive field and rapidly switches the polarization to the downward out-of-plane direction. This produces a net in-plane compressive strain in the PMN-PT substrate, which imposes a compressive stress on the SNO film and reduces its resistance. Upon releasing the field, a substantial residual strain remains, with the polarization predominantly aligned out-of-plane. By contrast, during the subsequent positive sweep from 0 to +1.8 kV/cm, the field remains below the coercive field and can only rotate part of the polarization from out-of-plane to in-plane, resulting in an in-plane tensile strain that imposes a tensile stress on the SNO film and increases its resistance. This non-180° polarization reorientation thus yields two distinct remanent resistance states at zero field, arising from the two significantly different strain states [19].

The inset of Figure 4a schematically illustrates the 71°, 109°, and 180° polarization switching paths in the PMN-PT (011) substrate [20]. Under symmetric bipolar electric fields, by contrast, the 109° and 180° switching events near the coercive fields (inset of Figure 3b) do not produce distinct remanent strain states because the associated domain states exhibit equivalent in-plane strain components. In the asymmetric case, however, the nonvolatile nature of ferroelastic domain switching locks in a remanent strain after the negative field is removed (from −7 to 0 kV/cm). As the positive field increases from 0 to +1.8 kV/cm, the downward out-of-plane polarization rotates in-plane, exerting an in-plane tensile stress on the SNO film and increasing its resistance. Because this positive field does not exceed the coercive field, full reversal to the upward out-of-plane state is prevented, enabling nonvolatile resistivity control, consistent with non-180° ferroelastic switching, including the 71° and 109° pathways reported for (011)-oriented PMN-PT. As demonstrated in Figure 4b, when asymmetric electric fields of different amplitudes are applied, the distinct residual strains under positive and negative fields lead to cyclic switching between two stable resistance states. The inset of Figure 4b illustrates the two corresponding polarization configurations: one with polarization pointing out of the plane (state ‘A’) and the other with polarization lying in the (011) plane (state ‘B’).

Preliminary retention (>12,000 s) and device-to-device reproducibility (three devices, ON/OFF ratio = 1.023 ± 0.003) confirm the nonvolatile nature of the switching (Figure S4). These proof-of-concept results establish the SNO/PMN-PT system as a promising platform, while extended endurance and statistical characterization remain for future work.

### 3.3. Thermally Enhanced Modulation Performance

**Figure 5:**
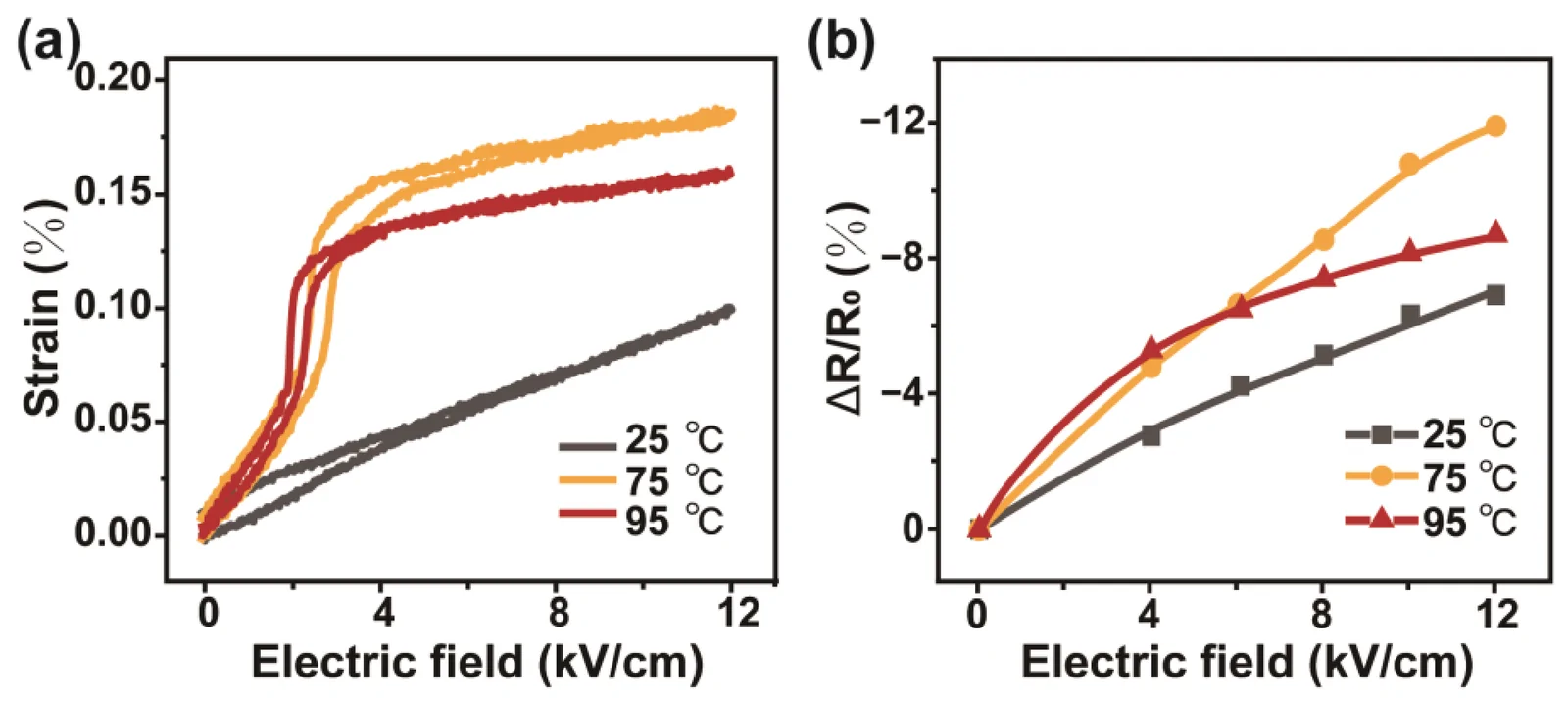
(**a**) Unipolar *ε*–*E* curves of (011)-oriented PMN-PT single crystal at different temperatures. (**b**) Temperature-dependent resistance response of SNO films under applied electric fields.

According to the Landau theory of phase transitions, temperature plays a decisive role in regulating the energy barrier for ferroelectric polarization rotation, especially near the critical phase boundary [21,22]. Figure 5a shows the *ε*–*E* curves of (011)-oriented PMN-PT single crystals at different temperatures. As the temperature increases from 25 °C to 75 °C, the field-induced strain increases significantly. Notably, the strain curve exhibits a nonlinear steep rise near 2.5 kV/cm. At 95 °C, this nonlinear region shifts to below 2 kV/cm, and the overall strain remains substantially higher than that at room temperature, though slightly lower than that at 75 °C.

The nonlinear electrostrain response observed at elevated temperatures is consistent with the well-documented phase transition behavior of (011)-oriented 0.70PMN-0.30PT near the morphotropic phase boundary (MPB) [23]. As reported, for the <011>-oriented crystal, an electric-field-dominated rhombohedral–orthorhombic (R–O) phase transition occurs at low temperatures, with the electrostrain peak shifting from 90 °C as the electric field increases. Under a small field (*E* ≤ 10 kV/cm), the electrostrain increases linearly with electric field due to polarization rotation within the R phase. In the quasi-MPB region, the combined effect of temperature and electric field can induce a series of phase transitions and polarization rotations, giving rise to large electrostrain [22,24].

In our *ε*–*E* curves (Figure 5a), at 25 °C and under fields up to 12 kV/cm, the electrostrain increases linearly with the electric field and remains relatively small, as the response is dominated by polarization rotation within the R phase. With increasing temperature, the steep strain increase near 2.5–3 kV/cm at 75 °C and the reduced critical field at 95 °C are consistent with this R–O transition behavior. The onset of this R–O phase transition leads to a marked enhancement of the electrostrain [25]. At 95 °C, the strain saturation above the critical field suggests that the response is dominated by polarization rotation within the O phase rather than by a phase transition itself.

Based on the temperature-dependent *ε*–*E* behavior of the PMN-PT substrate, we measured the strain-modulated resistance of the SNO film at different temperatures. As shown in Figure 5b, the enhanced out-of-plane tensile strain at elevated temperatures imposes an in-plane compressive strain on the SNO film, further reducing its resistance. At room temperature, a field of 12 kV/cm decreases the SNO resistance by −6.4%; at 75 °C and 95 °C, the resistance modulations reach −11.9% and −8.4%, respectively. At 75 °C, even under only 6 kV/cm, the modulation reaches −6.7%, exceeding the room-temperature value at 12 kV/cm. This temperature-dependent trend is consistent with the substrate strain behavior shown in Figure 5a. It is worth noting that at temperatures above 95 °C, the PMN-PT tends to transform into the tetragonal phase, which has a higher phase transition barrier. This likely causes the strain enhancement to saturate or increase more slowly at higher temperatures.

It is important to distinguish the intrinsic thermal effect on the SNO resistance from the strain-mediated modulation. At zero electric field, the SNO film exhibits a temperature-dependent resistance characterized by a negative temperature coefficient of resistance in the insulating regime. To eliminate the intrinsic thermal contribution to the resistance of the SNO film and isolate the strain-mediated effect, the resistance modulation ratio at each temperature is defined as Δ*R*/*R*_0_ = [*R*(*E*) − *R*(0)]/*R*(0), where *R*(0) represents the stable zero-field resistance measured at the same temperature. This normalization ensures that any variation in the modulation amplitude directly reflects the temperature-dependent strain transfer efficiency from the PMN-PT substrate, rather than the intrinsic temperature coefficient of resistance of the SNO film. In fact, while elevated temperature weakens the Jahn-Teller distortion [19], the highly distorted NiO_6_ octahedra themselves are intrinsically more responsive to external strain. To distinguish between substrate-strain enhancement and film-sensitivity change, we compared Δ*R*/*R*_0_ with strain at each temperature (Figure S5). The apparent strain sensitivity of SNO (defined as Δ*R*/*R*_0_ per unit substrate strain, extracted from the Δ*R*/*R*_0_–*E* and *ε*–*E* curves) is highest at 25 °C, followed by 95 °C, and lowest at 75 °C, indicating that the thermally boosted modulation originates primarily from the enhanced strain output of the PMN-PT substrate rather than from increased sensitivity of the SNO film.

To contextualize our results, we compare the strain-mediated resistive switching performance of our SNO/PMN-PT heterostructure with representative systems reported in the literature (Table 1). While NNO/PMN-PT achieves a larger modulation (125%), it operates at cryogenic temperatures (~200 K) due to the low MIT of NNO [14]. NiO/PMN-PT exhibits a comparable modulation of 82% at room temperature, but only in a volatile mode [26]. Our SNO/PMN-PT heterostructure uniquely combines (i) room-temperature to elevated-temperature, (ii) dual-mode (volatile + nonvolatile) switching within a single device, and (iii) a predominantly strain-mediated mechanism. These features establish SNO/PMN-PT as a distinct platform for strain-controlled resistive switching.

### 3.4. Limitations of the Study and Future Perspectives

In our SNO system, the thermally boosted, apparent strain-driven modulation amplitude of 11.9%—achieved in a lower-sensitivity insulating state—indicates the effectiveness of ferroelastic strain transfer and highlights the strong strain-electron coupling inherent to this system. While the modulation amplitude is moderate compared to NNO-based systems operating near their MIT critical point, the room-temperature operation and strain-mediated mechanism offer distinct advantages for practical device applications. Notably, the thermal enhancement allows for a ~2-fold increase in modulation amplitude at a significantly reduced electric field (6 kV/cm vs. 12 kV/cm), suggesting that mild heating could serve as a strategy to lower the operating voltage for neuromorphic-related applications. Direct temperature-dependent structural or dielectric characterization would provide more definitive confirmation of the R–O and O–T phase assignments, and such measurements remain a goal for future work.

As a proof-of-concept study, this work focuses on the demonstration of the strain-mediated dual-mode switching mechanism and the thermal enhancement effect. The retention (>12,000 s) and endurance (30 cycles) data provide preliminary evidence of nonvolatility and repeatability, sufficient for mechanism validation. Future device-level optimization should address extended cycling and longer retention to establish long-term reliability. Additionally, strategies such as tuning the SNO ground state closer to the MIT boundary through stoichiometry control, employing higher-performance piezoelectric substrates (e.g., PIN-PMN-PT) with larger strain coefficients, and constructing self-supporting heterojunction structures to release substrate clamping effects could further enhance the modulation amplitude and ON/OFF ratio [30]. We note that normalization by *R*(0) removes the baseline temperature dependence but does not by itself prove that the enhanced modulation originates exclusively from substrate strain. Direct strain-transfer measurements (e.g., in situ XRD on the SNO film) were not performed in this study, and remain a goal for future work. Further work may also focus on integrating these heterostructures onto silicon substrates, exploring faster switching speeds, and scaling down device dimensions for practical neuromorphic computing architectures [31].

## 4. Conclusions

In summary, we have achieved reversible and nonvolatile resistivity tuning in epitaxial SNO films through ferroelectric and ferroelastic domain engineering on (011)-cut PMN-PT substrates. Under symmetric bipolar electric fields, the heterostructure exhibits characteristic butterfly-shaped resistance hysteresis, consistent with electrostrain-mediated dynamic modulation. Nonvolatile resistance modulation is also achieved via non-180° ferroelastic domain switching, yielding a modulation ratio of 3.3% at room temperature. More importantly, thermally activated phase transition in the PMN-PT substrate significantly enhances the field-induced strain, boosting the modulation performance. At 75 °C, the resistance modulation increases from −6.4% (at 12 kV/cm, room temperature) to −11.9%; at 6 kV/cm, the modulation reaches −6.7%, exceeding that at 12 kV/cm under room-temperature conditions. The nonvolatile nature, combined with thermal enhancement, highlights the potential of SNO/PMN-PT heterostructures for strain-mediated resistive switching. Furthermore, the predominantly strain-mediated approach offers a physical pathway for future exploration in neuromorphic and memory applications. While the current demonstration serves as a proof-of-concept for strain-mediated dual-mode switching in the SNO/PMN-PT system, further device optimization—including extended endurance, switching speed, and energy consumption—is required for practical neuromorphic applications.

## Figures and Tables

**Table 1:** Comparison of strain-mediated resistive switching in representative oxide/ferroelectric heterostructures.

System	Orientation	Modulation	Operation T	Switching Mode	Ref.
La_0.6_Sr_0.4_MnO_3_/PMN-PT	(011)	3.6%	RT	Nonvolatile	[27]
NdNiO_3_/PMN-PT	(001)	1.5%	RT	Volatile	[13]
NdNiO_3_/PMN-PT	(011)	125%	200 K	Nonvolatile	[14]
NiO/PMN-PT	(001)	82%	RT	Volatile	[26]
Cr:In_2_O_3_/PMN-PT	(111)	0.5%	RT	Nonvolatile	[28]
NdNiO_3_/PMN-PT	(001)	4.5%	77 K	Volatile	[29]
SmNiO_3_/PMN-PT (this work)	(011)	6.4%~11.9%	RT to 348 K	Volatile + Nonvolatile	

## Data Availability

The original contributions presented in this study are included in the article/Supplementary Material. Further inquiries can be directed to the corresponding author.

## Supplementary Materials

The following supporting information can be downloaded at: https://www.mdpi.com/article/10.3390/ma19183838/s1. Figure S1: Cross-sectional SEM images of representative calibration films of (**a**) SrTiO_3_ (STO) and (**b**) LaAlO_3_ (LAO) grown under identical deposition conditions for 60 minutes. The estimated deposition rates of STO and LAO are approximately 5.0 and 5.2 nm/min, respectively, corresponding to buffer layer thicknesses of about 5 nm for the 1-minute deposition used in the heterostructure, Figure S2: Schematic illustration of the device configuration and electrical measurement setup (Inset: optical microscopy image of Pt on SNO film). The electric field is applied out-of-plane across the SNO/PMN-PT heterostructure via Pt top electrodes (~200 μm diameter, deposited on SNO) and an Au bottom electrode (sputtered on the backside of the PMN-PT substrate). The in-plane resistance of the SNO film is measured in a two-terminal configuration with a probe spacing of approximately 300 μm. The resistance measurement current path (100 µA) and the out-of-plane electric field direction are indicated, Figure S3: Polarization–electric field (*P*–*E*) hysteresis loop of the PMN-PT (011) substrate measured under symmetric bipolar electric fields at room temperature. The rectangular/square-like hysteresis is characteristic of ferroelectric polarization switching. The coercive field is approximately 2.0 kV/cm, consistent with the resistance peaks in our Δ*R*/*R*_0_–*E* curves (~2 kV/cm), confirming that the resistance change is strain-mediated, Figure S4: Nonvolatile and reproducible switching characteristics of SNO/PMN-PT devices. (**a**) Retention stability of the high-resistance state (*R*_H_, at +0 kV/cm) and low-resistance state (*R*_L_, at −0 kV/cm) measured at room temperature for >12,000 s under zero electric field. (**b**) Endurance performance over 30 consecutive switching cycles, demonstrating a stable ON/OFF ratio (defined as *R*_H_/*R*_L_) of 1.023 ± 0.003. (**c**,**d**) Reproducibility test of two additional independent devices from the same batch, showing consistent switching behavior across devices, Figure S5: Apparent/effective strain response of the SNO film relative to the substrate strain (Δ*R*/*R*_0_ per unit strain) at 25 °C, 75 °C, and 95 °C, extracted from the *ε*–*E* and Δ*R*/*R*_0_–*E* curves. The apparent sensitivity follows the order 25 °C > 95 °C > 75 °C, indicating that the thermally enhanced modulation is primarily driven by substrate strain enhancement rather than film sensitivity change.
