# Supplementary material for: Thermally Enhanced Dual-Mode Resistive Switching via Interfacial Strain Engineering in SmNiO3/PMN-PT Heterostructures
Source: Materials (Basel). 2026 Sep 9;19(18):3838. doi: 10.3390/ma19183838 (PMC13608159; doi:10.3390/ma19183838)
Supplement: Supplementary file 1 [file materials-19-03838-s001.zip › materials-4504273-supplementary.pdf]

Supplementary Data

Thermally Enhanced Dual-Mode Resistive Switching via Interfacial Strain Engineering in $SmNiO_3$/PMN-PT Heterostructures

Jianfeng Yang and Qin Du *

Shaanxi Key Laboratory of Intelligent Processing for Big Energy Data, School of Physics and Electronic Information, Yan'an University, Yan'an 716000, China; yangjf@yau.edu.cn
* Correspondence: duqin0422@yau.edu.cn

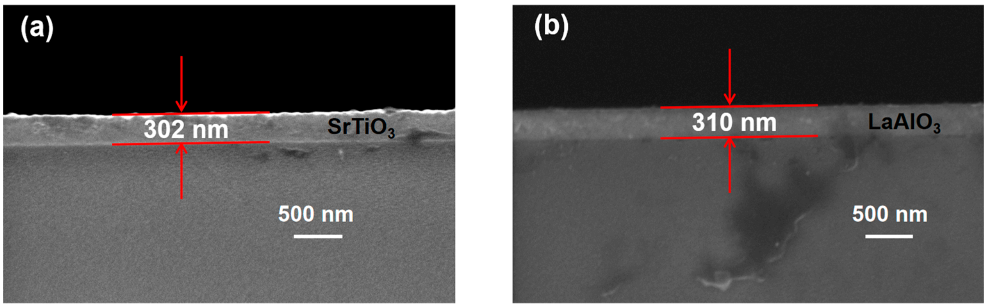

Figure S1. Cross-sectional SEM images of representative calibration films of (a) $SrTiO_3$ (STO) and (b) $LaAlO_3$ (LAO) grown under identical deposition conditions for 60 minutes. The estimated deposition rates of STO and LAO are approximately 5.0 and 5.2 nm/min, respectively, corresponding to buffer layer thicknesses of about 5 nm for the 1-minute deposition used in the heterostructure.

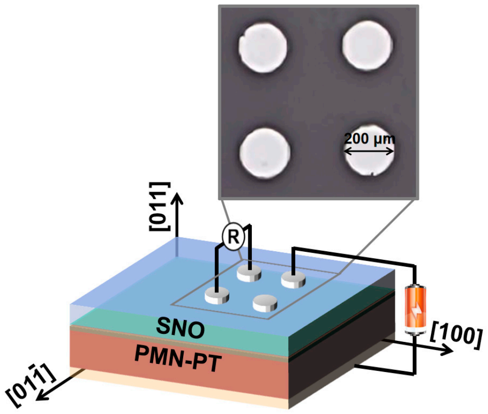

Figure S2. Schematic illustration of the device configuration and electrical measurement setup (Inset: optical microscopy image of Pt on SNO film). The electric field is applied out-of-plane across the SNO/PMN-PT heterostructure via Pt top electrodes (~200 μm diameter, deposited on SNO) and an Au bottom electrode (sputtered on the backside of the PMN-PT substrate). The in-plane resistance of the SNO film is measured in a two-terminal configuration with a probe spacing of approximately 300 μm. The resistance measurement current path (100 μA) and the out-of-plane electric field direction are indicated.

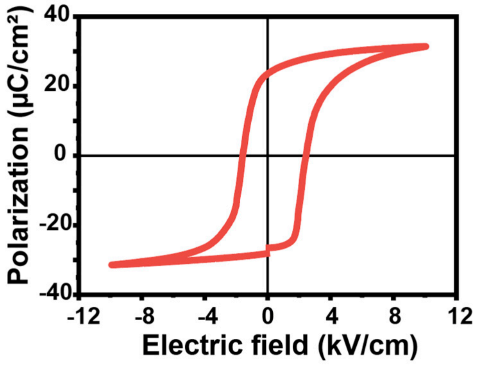

Figure S3. Polarization–electric field (*P*–*E*) hysteresis loop of the PMN-PT (011) substrate measured under symmetric bipolar electric fields at room temperature. The rectangular/square-like hysteresis is characteristic of ferroelectric polarization switching. The coercive field is approximately 2.0 kV/cm, consistent with the resistance peaks in our $\Delta R/R_0$–E curves (~2 kV/cm), confirming that the resistance change is strain-mediated.

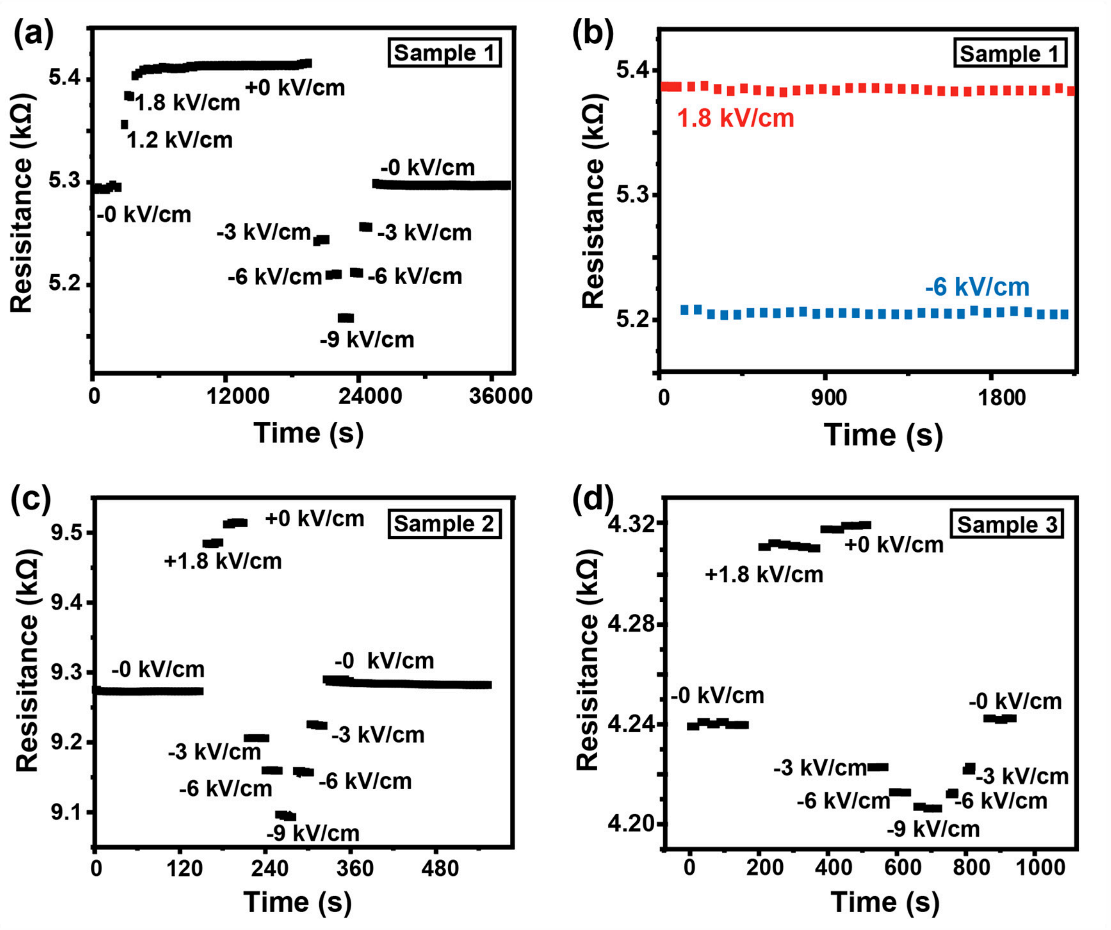

Figure S4. Nonvolatile and reproducible switching characteristics of SNO/PMN-PT devices. (a) Retention stability of the high-resistance state ($R_{H,}$ at +0 kV/cm) and low-resistance state ($R_{L,}$ at -0 kV/cm) measured at room temperature for >12,000 s under zero electric field. (b) Endurance performance over 30 consecutive switching cycles, demonstrating a stable ON/OFF ratio (defined as R_H/R_L) of 1.023 ± 0.003. (c, d) Reproducibility test of two additional independent devices from the same batch, showing consistent switching behavior across devices.

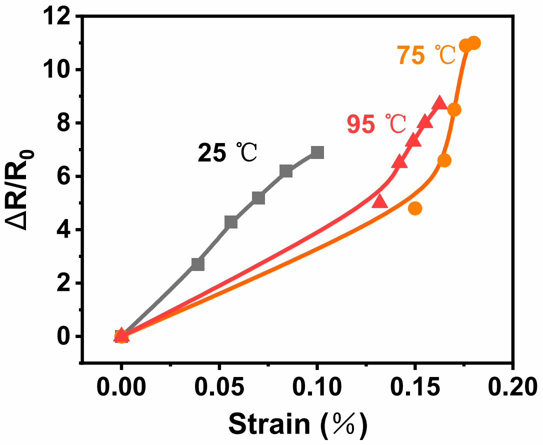

Figure S5. Apparent/effective strain response of the SNO film relative to the substrate strain ($\Delta R/R_0$ per unit strain) at 25 °C, 75 °C, and 95 °C, extracted from the ε–E and $\Delta R/R_0$–E curves. The apparent sensitivity follows the order 25 °C > 95 °C > 75 °C, indicating that the thermally enhanced modulation is primarily driven by substrate strain enhancement rather than film sensitivity change.
